# Giant Right Coronary Artery Aneurysm Presenting as Syncope

**DOI:** 10.7759/cureus.101684

**Published:** 2026-01-16

**Authors:** Marios-Vasileios Koutroulos, Georgios Chalikias, Dimitrios Tziakas

**Affiliations:** 1 Department of Cardiology, University General Hospital of Alexandroupolis, Alexandroupolis, GRC

**Keywords:** a coronary artery anomaly, coronary angiography, coronary artery aneurysm, right coronary artery (rca), syncope

## Abstract

Coronary artery aneurysms (CAAs) are rare findings, most commonly detected incidentally during coronary angiography or advanced cardiac imaging. Giant aneurysms of the right coronary artery are exceptionally uncommon and may lead to severe complications, including thrombosis, embolization, myocardial infarction, or sudden cardiac death. We present the case of a 78-year-old male who was diagnosed with a giant right CAA during evaluation for syncope. The patient underwent successful surgical excision of the aneurysm with revascularization of the right coronary artery. This case highlights the diagnostic value of coronary angiography and the role of surgical management in large CAAs.

## Introduction

Coronary artery aneurysm (CAA) is defined as a localized dilatation of a coronary artery segment exceeding 1.5 times the diameter of the adjacent normal vessel. CAAs are relatively rare, reported in approximately 0.5-5% of patients undergoing coronary angiography. The right coronary artery is the most frequently affected vessel.

The etiology of CAAs includes both congenital and acquired causes. In adults, atherosclerosis represents the most common underlying mechanism, whereas Kawasaki disease is the predominant cause in pediatric populations. Additional etiologies include inflammatory vasculitides, connective tissue disorders, infections, and iatrogenic injury following coronary interventions.

Giant CAAs, generally defined as aneurysms exceeding 20-30 mm in diameter, are exceedingly rare and carry a significantly worse prognosis. Management strategies are not standardized and depend on aneurysm size, location, symptoms, and associated complications. Surgical treatment is often preferred for large or complex aneurysms.

## Case presentation

A 78-year-old male was transferred from a regional hospital following hospitalization for a syncopal episode. During the initial evaluation at the referring hospital, imaging findings raised suspicion of a large coronary abnormality, prompting transfer to our tertiary center for further assessment.

According to the available documentation from the referring hospital, the syncopal episode was sudden, without preceding chest pain or palpitations, and resolved spontaneously. Initial evaluation included a 12-lead electrocardiogram, which showed no acute ischemic changes or significant conduction abnormalities. Continuous cardiac telemetry did not reveal sustained arrhythmias. Routine laboratory testing was unremarkable. The initial diagnostic workup did not identify a clear cause of syncope, and imaging performed at the referring hospital raised suspicion of a significant coronary abnormality, prompting transfer to our tertiary center for further evaluation.

The patient was referred to the University General Hospital of Alexandroupolis for further evaluation. Upon admission to the coronary care unit, he was hemodynamically stable and asymptomatic, with no chest pain or signs of heart failure.

**Figure 1:**
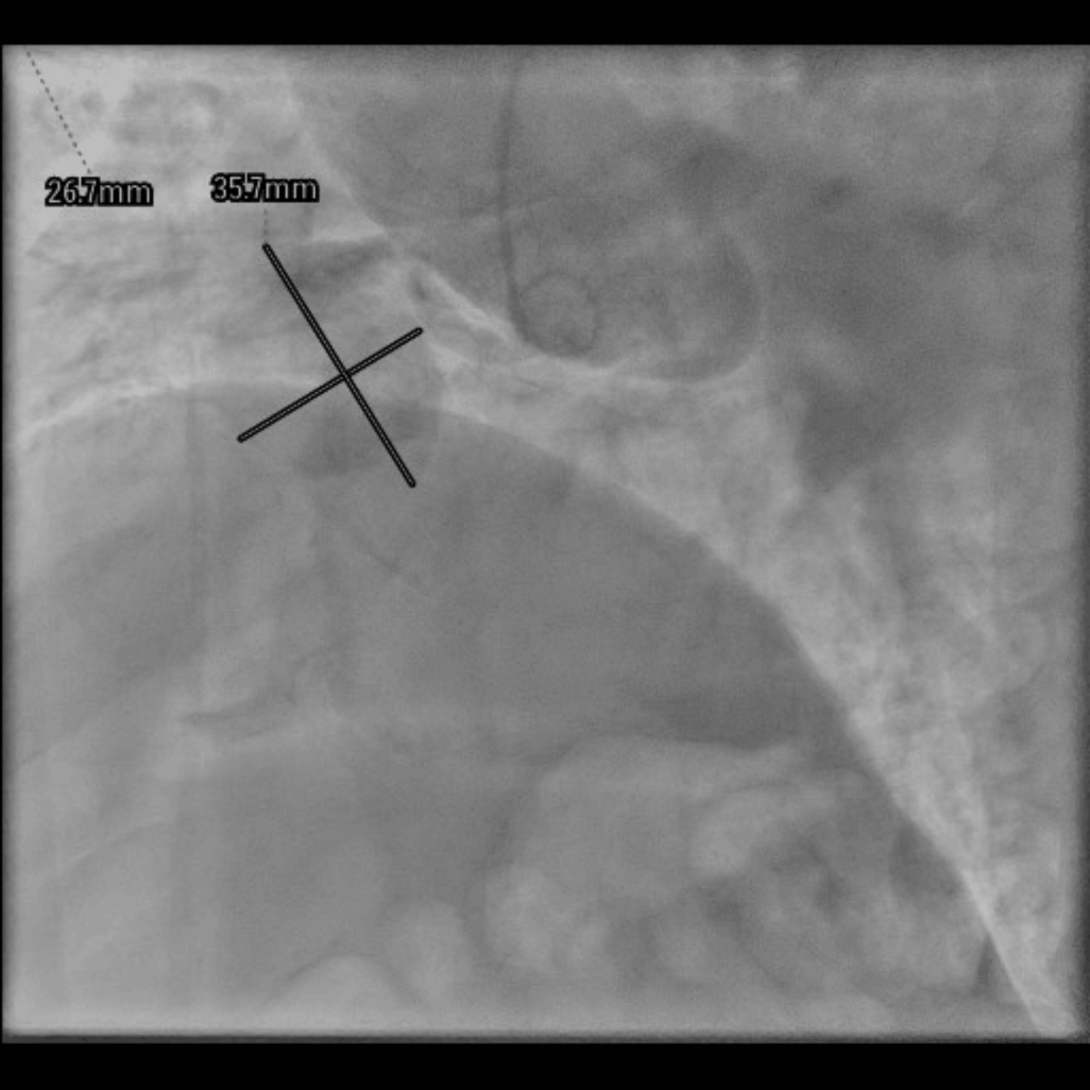
Coronary angiography revealing a giant aneurysm of the right coronary artery

The patient underwent urgent coronary angiography, which confirmed the presence of a giant aneurysm of the right coronary artery, measuring approximately 3.5 × 2.5 cm, based on angiographic measurements (Figure 1). Coronary angiography remains a cornerstone in the diagnosis of CAAs, allowing assessment of aneurysm morphology and distal vessel patency [1,2].

**Figure 2:**
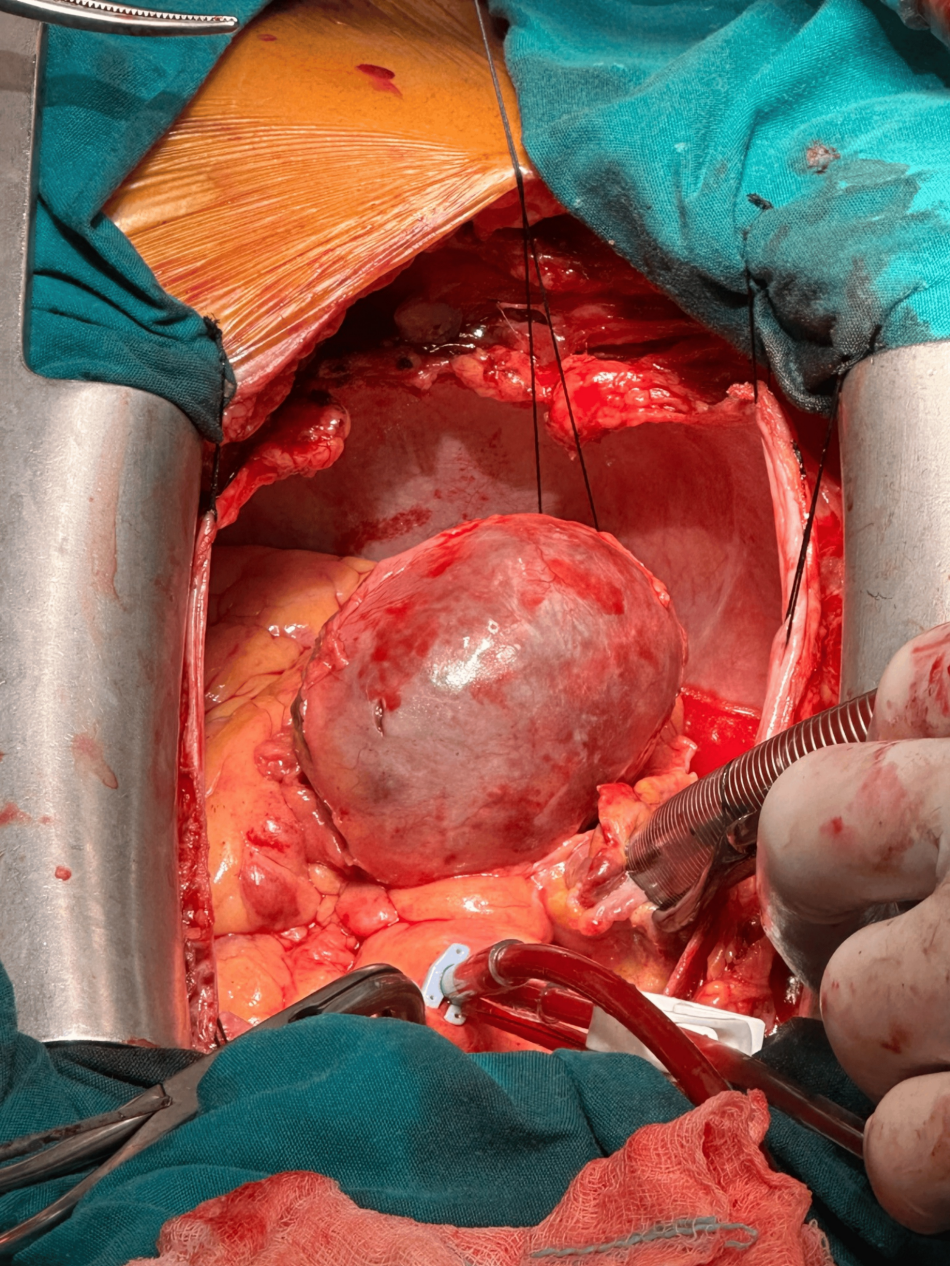
Surgical excision of the aneurysmal sac

Given the aneurysm’s size and the increased risk of thromboembolic and compressive complications, a multidisciplinary decision was made to proceed with surgical management. The patient was transferred to the cardiothoracic surgery department, where surgical excision of the aneurysmal sac was performed, followed by surgical revascularization of the right coronary artery (Figure 2).

**Figure 3:**
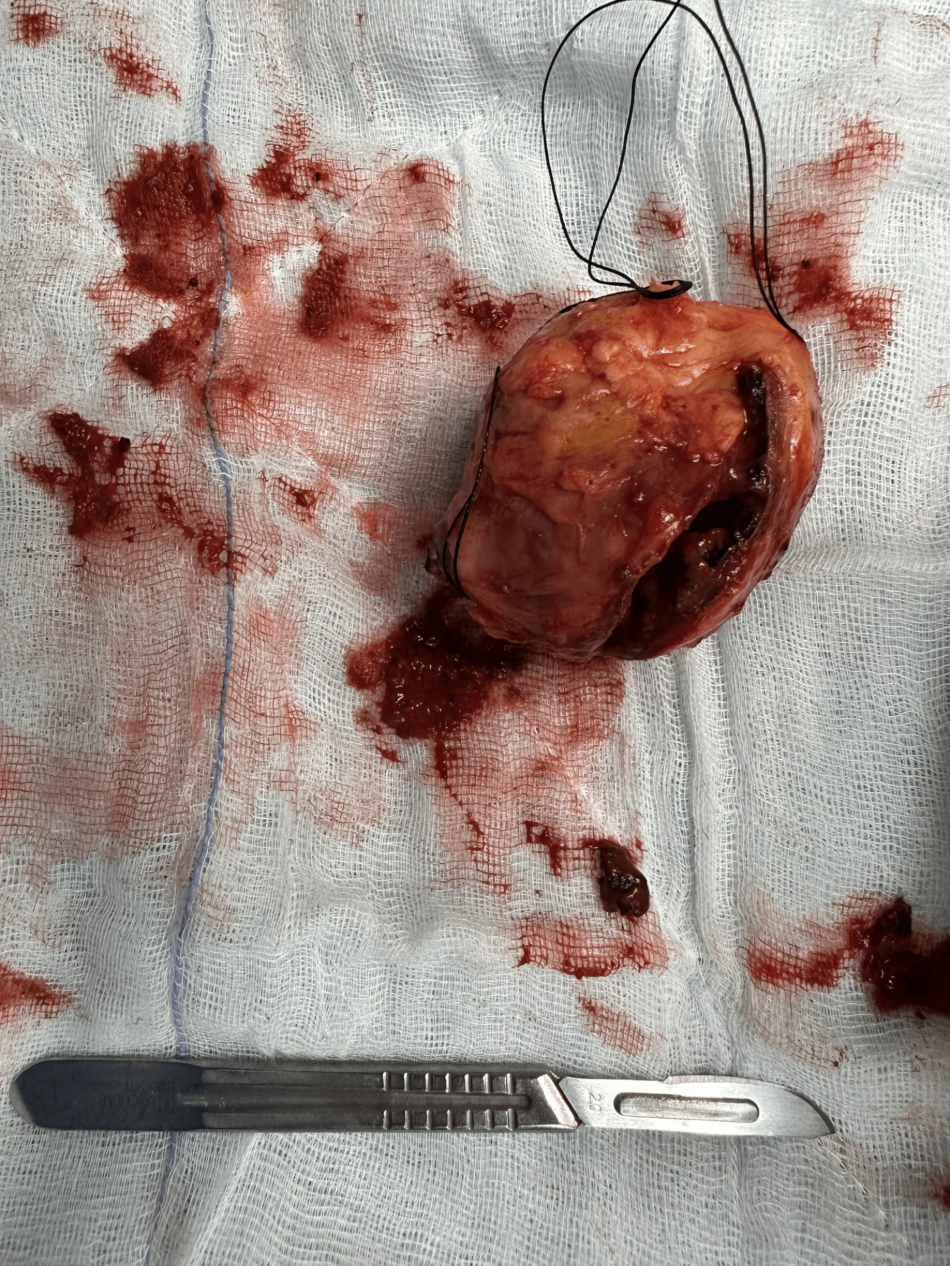
Excised aneurysmal specimen

The excised aneurysmal specimen is shown in Figure 3. The postoperative course was uneventful, and the patient recovered without complications.

## Discussion

Giant aneurysms of the right coronary artery represent a rare but clinically significant entity. While many CAAs remain asymptomatic, giant aneurysms are associated with an increased risk of serious complications, including thrombosis, distal embolization, myocardial ischemia, rupture, and compression of adjacent cardiac structures [1,3,4].

Although coronary angiography is essential for defining coronary anatomy, it may underestimate the true size of aneurysms due to partial thrombosis of the aneurysmal sac [5]. Cross-sectional imaging modalities, such as CT coronary angiography, may provide additional anatomical detail; however, angiography alone can be sufficient to guide management decisions in selected cases [2].

While percutaneous treatment options, including covered stent implantation or coil embolization, have been described in selected patients, surgical intervention remains the preferred treatment for giant CAAs, particularly in cases with large aneurysms or high complication risk [6-8]. Surgical strategies typically include aneurysm ligation or resection combined with coronary artery bypass or revascularization, as performed in our patient [9].

This case emphasizes the importance of considering rare coronary pathologies in the differential diagnosis of syncope and supports the role of timely surgical management in achieving favorable outcomes.

CAAs represent a heterogeneous clinical entity with variable etiologies, morphologies, and clinical presentations. While many cases are detected incidentally, giant aneurysms are associated with an increased risk of thrombus formation, distal embolization, myocardial ischaemia, rupture, and compression of adjacent cardiac structures. The right coronary artery is most commonly involved, and aneurysm size remains one of the strongest predictors of adverse outcomes. Due to the rarity of giant CAAs, evidence-based management guidelines are lacking, and treatment decisions are typically individualized, taking into account aneurysm size, location, symptoms, and patient comorbidities. In this context, surgical management remains the preferred strategy for large or high-risk aneurysms [1,9].

## Conclusions

Giant right coronary artery aneurysms are rare and potentially life-threatening conditions. Coronary angiography plays a key role in diagnosis, while surgical excision with revascularization remains the treatment of choice in large aneurysms. Early recognition and a multidisciplinary approach are essential to prevent serious complications.
